# Research and Technological Advances Regarding the Study of the Spread of Antimicrobial Resistance Genes and Antimicrobial-Resistant Bacteria Related to Animal Husbandry

**DOI:** 10.3390/ijerph16244896

**Published:** 2019-12-04

**Authors:** Na Li, Chong Liu, Zhiguo Zhang, Hongna Li, Tingting Song, Ting Liang, Binxu Li, Luyao Li, Shuo Feng, Qianqian Su, Jing Ye, Changxiong Zhu

**Affiliations:** Institute of Environment and Sustainable Development in Agriculture, Chinese Academy of Agricultural Sciences, Beijing 100081, China; 82101171047@caas.cn (N.L.); liuchong@caas.cn (C.L.); zzg18615535143@163.com (Z.Z.); lihongna@caas.cn (H.L.); songtingting0505@163.com (T.S.); 15736873151@163.com (T.L.); libinxu123@163.com (B.L.); liluyao0215@163.com (L.L.); 82101185092@caas.cn (S.F.); 15234170575@163.com (Q.S.); yejing@caas.cn (J.Y.)

**Keywords:** animal husbandry, antimicrobials, antibiotic resistance genes, pathogen, high-throughput sequencing, metagenomic analysis

## Abstract

The extensive use of antimicrobials in animal farms poses serious safety hazards to both the environment and public health, and this trend is likely to continue. Antimicrobial resistance genes (ARGs) are a class of emerging pollutants that are difficult to remove once introduced. Understanding the environmental transfer of antimicrobial-resistant bacteria (ARB) and ARGs is pivotal for creating control measures. In this review, we summarize the research progress on the spread and detection of ARB and ARG pollution related to animal husbandry. Molecular methods such as high-throughput sequencing have greatly enriched the information about ARB communities. However, it remains challenging to delineate mechanisms regarding ARG induction, transmission, and tempo-spatial changes in the whole process, from animal husbandry to multiple ecosystems. As a result, future research should be more focused on the mechanisms of ARG induction, transmission, and control. We also expect that future research will rely more heavily on metagenomic -analysis, metatranscriptomic sequencing, and multi-omics technologies

## 1. Introduction

Intensive farming and animal husbandry have developed tremendously in many parts of the world over the past five decades. Global meat production has almost quadrupled from 84 million tons in 1965 to about 335 million tons in 2018, and this trend will most likely continue [1]. Intensive animal husbandry is not a global practice but rather is region-dependent. China and the United States are currently the largest global producers of meat and animal products [2]. Animal farms are zoonotic pathogen reservoirs, as well as sources of veterinary antimicrobials and antimicrobial resistance genes (ARGs). The demand for veterinary antimicrobials has risen sharply over the past few decades due to the development of intensive livestock farming. This is especially the case in countries where antimicrobial manufacturing occurs on a substantial scale but lacks essential regulation [3]. Application of animal manure to agricultural soils and leakage from manure lagoons have been regarded as the primary antimicrobial-resistant bacteria (ARB) sources at the farm level [4]. On livestock farms with modern environmental protection equipment, chemical pollutants such as ammonia, methane, chemical oxygen demand (COD), and total nitrogen and phosphorus can be reduced by anaerobic fermentation and treatment of manure and wastewater. The manure can be used as organic fertilizer to recycle resources, but with a caveat—harmful microorganisms and ARGs are often difficult to remove. Although regional legislation may restrict terrestrial application of manures due to the presence of transmissible disease organisms, antimicrobial resistance is currently not considered [5]. At present, numerous studies have addressed the spread of microbial pathogens and ARGs caused by animal husbandry, including the production and spread of antimicrobial-resistant bacteria (ARB).

The use of large quantities of antimicrobials is one of the factors that influence ARB and ARG contamination on animal farms. In many countries, antimicrobials are used for disease treatment and prevention, as well as to promote animal growth and fattening. The quantity of antimicrobials added to animal feed ranges from 3–220 g/t and is dependent on animal species and antimicrobial type [6]. As feed additives, antimicrobials can exert selective pressure on animal intestinal bacteria, resulting in ARB proliferation that is then discharged into the environment through excretion [7]. Animal-derived pathogens can also easily assimilate ARGs that can then be transferred to humans by contact or consumption of raw fruits and vegetables containing these pathogens; this poses a threat to public health [8]. Even the most advanced wastewater treatment procedures cannot remove all ARB before discharge [9,10,11,12]. The associated risks and mechanisms of ARG transmission, transmission pathways, and technical methods have been studied with fruitful results being achieved, but there are still many unknown factors.

In brief, animal husbandry can cause ARB and ARG pollution in the environment and is a pressing problem for animal husbandry operations. This paper reviews the research progress of ARB and ARG pollution related to animal husbandry, including the application status of antimicrobials in animal husbandry, the mechanism of ARG induction, and technological research methods of ARGs and ARB. On this basis, we discussed the emerging trends of research and technological methods in this field towards the goals of demonstrating and removing ARG pollution caused by animal husbandry.

## 2. Methods

To illustrate how many articles have been published in the last five years, we searched on Web of Science for the techniques used to study antimicrobial resistance genes. These advanced search queries are listed below. There were five searches for each query based on the year for the following search queries, with each search changing PY (published year) to a different year from 2014 to 2018.

### 2.1. Metagenomic Analysis

TS = ((ARGs OR ARG OR “anti* resistance gene*”) AND "metagenom*") AND PY = 2014. In this query, TS means topic searches, the same below.

### 2.2. Animal Husbandry Related Metagenomic Analysis

TS = ((ARGs OR ARG OR “anti* resistance gene*”) AND "metagenom*" AND (pig* OR swine OR hog* OR chicken OR broiler* OR layer* OR pigeon* OR dove* OR turkey* OR duck* OR sheep Or goat OR dairy OR bovine OR cow OR cattle* OR feedlot* OR livestock OR poultry OR manure)) AND PY = 2014.

### 2.3. Quantitative PCR (qPCR)

TS = ((ARGs OR ARG OR “anti* resistance gene*”) AND ("qPCR" OR "quantitative p*" NOT “high-throughput q*” NOT “high-capacity q*”)) AND PY = 2014.

### 2.4. Animal Husbandry Related qPCR

TS = ((ARGs OR ARG OR “anti* resistance gene*”) AND ("qPCR" OR " quantitative p*" NOT “high-throughput q*” NOT “high-capacity q*”) AND (pig* OR swine OR hog* OR chicken OR broiler* OR layer* OR pigeon* OR dove* OR turkey* OR duck* OR sheep OR goat OR dairy OR bovine OR cow OR cattle* OR feedlot* OR livestock OR poultry OR manure)) AND PY = 2014.

### 2.5. High-Throughput qPCR

TS = ((ARGs OR ARG OR “anti* resistance gene*”) AND (“high-throughput quantitative” OR “high-capacity quantitative”)) AND PY = 2014.

### 2.6. Animal Husbandry Related High-Throughput qPCR

TS = ((ARGs OR ARG OR “anti* resistance gene*”) AND (“high-throughput quantitative” OR “high-capacity quantitative”) AND (pig* OR swine OR hog* OR chicken OR broiler* OR layer* OR pigeon* OR dove* OR turkey* OR duck* OR sheep OR goat OR dairy OR bovine OR cow OR cattle* OR feedlot* OR livestock OR poultry OR manure)) AND PY = 2014.

### 2.7. -16S rDNA Taxonomic Composition

TS = ((ARGs OR ARG OR “anti* resistance gene*”) AND (“high-throughput sequencing” OR “next-generation sequencing” OR “next-generation 16S”)) AND PY = 2014.

### 2.8. Animal-Husbandry-Related 16S rDNA Taxonomic Composition

TS = ((ARGs OR ARG OR “anti* resistance gene*”) AND (“high-throughput sequencing” OR “next-generation sequencing” OR “next-generation 16S”)AND (pigs OR swine OR hog* OR chicken OR broiler* OR layer* OR pigeon* OR dove* OR turkey* OR duck* OR sheep OR goat OR dairy OR bovine OR cow OR cattle* OR feedlot* OR livestock OR poultry OR manure)) AND PY = 2014.

## 3. The State of Current Veterinary Research of ARB and ARG Pollution

### 3.1. Impact of Veterinary Antimicrobials on ARB and ARGs, and Their Correlation with ARGs

The large-scale production and use of antimicrobials is a key factor in bacterial resistance. The European Union and the United States have taken measures to limit antimicrobial use, such as forbidding antimicrobials as additives to promote animal growth and fattening. Globally, however, the use of antimicrobials has been increasing [13]. In China for example, antimicrobials have been used as low-dose feed additives for livestock and poultry since only the mid-1970s. Nonetheless, China is currently the global leader in production and consumption of antimicrobials for animals [14,15]. A new published report showed that antimicrobial usage for animal husbandry in China reduced from the year 2014 to 2018, with 29,774 tons used in 2018 [16]. Meanwhile, the good news is that The Ministry of Agriculture and Rural Affairs of the People’s Republic of China has set a new regulation, which will restrict the use of antimicrobials as animal feed additives aimed at promoting animal growth from January 2020 [17]. In the United States, food animals account for about 80% of the total domestic consumption of antimicrobials [18]. In many low- and middle-income countries, antimicrobial use is rapidly increasing because of the escalating demand for animal protein, shifting animal husbandry into largescale intensive operations. For example, in Brazil, Russia, India, China, and South Africa (BRICS countries), the antimicrobial consumption is estimated to increase by 99% from 2010 to 2030, up to seven times the predicted population growth in these countries in the same period [19].

The fate of antimicrobials discharged into the environment is complex, as the degree of antimicrobial adsorption in soil depends on the antimicrobial type, soil properties [20], and microbial processes [21]. For example, the amount of tetracyclines present in pig manure could be reduced by composting, but soil fertilized with the compost still contains up to 776.1 μg/kg and is considered sufficient for an ARB-selective effect [22]. Antimicrobials entering the soil can be altered by adsorption, photolysis, hydrolysis, and microbial degradation [23]. However, the tetracyclines can be adsorbed tightly into soil particles and resist biodegradation [24], and some antimicrobials may persist in soil for years [25,26]. The degree of antimicrobial adsorption in soil depends on the antimicrobial type, soil properties [20], and microbial processes [21]. Some antimicrobials with short half-life and strong adsorption on soil particles are difficult to detect in water and plants, but those with long half-lives and those that can be adsorbed in soil particles may be taken up by plants [6]. Meanwhile, the antimicrobial level needed in a particular environmental matrix to cause selective effects was not clear, as it often depends on the antimicrobial class and even on individuals. Research studies have shown that sulfonamides in soils ≥ 0.1 mg/kg and ciprofloxacin in a liquid medium at 100 ng/L can induce ARB selection [27,28].

There is a background level of antimicrobial resistance that has evolved in bacteria in the environment due to selection pressure from competing organisms. Under natural conditions, a low level of antibiotics may promote bacterial growth, while high levels inhibit bacterial growth (the Hormesis effect) [29]. However, with their use and discharge in large quantities in recent decades, high levels of antimicrobials exert a dominant selective pressure. This includes ARG acquisition by non-resistant bacteria through horizontal gene transfer (HGT) and other mechanisms to increase ARB in the natural community structure. HGT is usually achieved through passage of mobile genetic elements (MGEs), such as plasmids, transposons, integrons, phages, and insertion sequences between species within a bacterial community. Plasmid-mediated conjugation is considered the most common HGT process, followed by transduction and transformation [30,31,32]. Meanwhile, bacteria express resistance via different mechanisms. Under this selective pressure, the primary mechanisms of resistance are through efflux pumps, enzyme inactivation, and cell protection [33]. Different ARGs may also possess different ecologies. Tetracycline resistance genes, ribosomal protection, and enzyme inactivation mechanisms are generally more frequently detected in manure and manure-applied soil, while the efflux pump genes are more frequently detected in prairie soil [34]. Perhaps one of the reasons is that efflux pumps are mainly chromosomally-encoded and present a conserved organization, while only a few are in MGEs [35]. A major concern is that bacterial ARGs persist even if antimicrobial use is stopped [36]. There is presently little information on the length of time ARGs persist because of the lack of an integrated accounting system for antimicrobial use on farms [4].

A World Health Organization (WHO) survey demonstrated that greater antimicrobial use at the national level leads to wider microbial resistance [37]. This seems especially true in some countries where antimicrobials are used more extensively than in other countries, among which China and India have been reported to have the most widespread antimicrobial resistance in livestock. In these countries, immediate actions are suggested to restrict antimicrobial use for animals [19]. Interestingly, this ranking is consistent with thier usage status of antimicrobials in animal husbandry [3]. Among other countries, Brazil and Kenya are emerging antimicrobial resistance hotspots [19].

The *mcr-1* gene, encoding colistin resistance, was first found in China in 2015 and is causing concern all over the world because colistin was considered the last resort for treating gram-negative bacterial infections. A 15% prevalence of *mcr*-1-positive isolates was found in Chinese retail meat, while only 1.5% and 2% prevalence were found in Dutch and Danish chicken meat, respectively. The latter were associated with low rates of polymyxin use in European livestock [38]. Currently, Asia and the Americas show the most severe colistin resistance in animal husbandry epidemic analysis, and regional spreading of colistin resistance may be driven by plasmid-mediated resistance [19]. Currently, a higher frequency of *mcr* genes have been reported in bacterial isolates from animals than humans. Pigs have been ascribed the most among animals for the spreading colistin-resistant bacteria [39]. On the other hand, sanitation products other than antimicrobials and heavy metals could also increase antimicrobial resistance levels [40], which could be another factor making it hard to control ARG induction and transfer in the antimicrobial-restricted countries who tried to reduce ARB pollution levels. International travelers from the Netherlands to some Asian countries exhibited the highest acquisition frequency of *qnrS* and both *blaCTX-M* and *qnrS*, respectively [41]. These factors contribute to making ARB and ARGs ubiquitous and persistent pollutants. ARB cannot be degraded but can auto-replicate and can be acquired by eating polluted foods, prolonged stays in clinical environments, and long-distance travel, and this acquisition is independent of prior antimicrobial exposure. 

In current epidemic data for animals, rates of resistance are dependent on animal class and region. For example, from 2000 to 2018, the proportion of antimicrobials showing rates of resistance above 50% in developing countries increased from 0.15 to 0.41 and 0.13 to 0.34 in chickens and in pigs, respectively. This means that antimicrobials that could be used for treatment failed more than half the time in 40 percent of chickens and 34 percent of pigs raised for human food [19]. Therefore, in some developing countries, stricter regulations regarding antimicrobial use in animal husbandry is suggested by researchers. As for animal classes, chicken and pigs have higher resistance rates than cattle, and the resistance rates of the former two animals have increased significantly over time since 2000, while cattle showed no significant change overtime [19]. This resistance order is also consistent with the antimicrobials currently used in these three species of animals. For instance, the average annual consumption of antimicrobials per kilogram of animal produced was 45, 148, and 172 mg·kg^−1^ for cattle, chicken, and pigs, respectively. Currently, the role and exchange of ARGs between humans and animals and the environment is not completely clear, although CTX-M-15, the most widely distributed cefotaximase-Munich (CTX-M)-type betalactamase between human *Enterobacteriaceae* has also been found in *Escherichia coli* from poultry and pigs [42]. 

Unlike livestock or poultry, there seems to be no linkage between antimicrobial use and ARB in aquaculture, as strains in aquatic animals may contain high levels of antimicrobial resistance, even if the animals have never been exposed to antimicrobials. Additionally, the microflora in aquatic animals change a lot after these animals have been handled and processed. Therefore, the microflora in farmed fish at the retail level do not necessarily represent the original ones in aquaculture environments. Antimicrobial resistance levels in aquatic bacteria can be very high as far as public health is concerned. For example, bacteria isolated from crustaceans and shellfish in China have 90% resistance to rifampin and 78% resistance to streptomycin [43].

In general, the correlation between antimicrobials and ARGs is complex; selective pressure caused by the presence of antimicrobials is not the only factor related to environmental ARG induction and persistence within microbes [44]. These other factors include the complexities of environmental matrices as well as other factors that are still not entirely defined [45,46]. Theoretically, any factor able to cause a microbial community shift will influence resistome composition at the community level. Despite the fact that the corresponding ARGs appear shortly after the induction of a novel antimicrobial in the market, we should note that ARGs are ancient and exist on a large scale [47]. For example, *Brevundimonas* possesses innate resistance to fluoroquinolones [40]. Fluoroquinolone resistance also exists in environmental samples that have never been exposed to antimicrobials [48]. *Pedobacter* spp. are environmental superbugs and likely intrinsically resistant to β-lactams, colistin, aminoglycosides, and ciprofloxacin [49]. ARGs such as *bla*_CTX−M_, *qnrA*, and *bla*_NDM_ are believed to have evolved in nature with unknown functions [50].

### 3.2. ARG Environmental Transfer

The natural background level of antimicrobials in soil is augmented on animal farms by contaminated manure and wastewater, which introduce foreign antimicrobials as well as resistant bacteria into the system. An examination of archived soils indicated that ARG abundance in soil bacteria has increased significantly since antimicrobials were first produced and used in large quantities in the 1940s [51]. In water ecosystems, ARB wastewater and pollution from farms and sewage treatment plants can pollute rivers and groundwater. For instance, antimicrobial type and dosage have been implicated in defining the ARG composition in pig wastewater microbiota [52]. Groundwater can be easily polluted by antimicrobials and ARB in the surrounding environment due to the penetration of surface water and soil materials. Harmful microorganisms and ARG pollution can also be detected in the aerosols inside and around farms, because the activities of animals and the treatment of manure can cause microorganisms to diffuse into the air to form biological aerosols. Bacteria aerosolized from animal farm feces can reach levels as high as 10^5^ CFU/m^3^ [53]. Ventilation and air flow also assist in the spread of these harmful aerosols that can settle on vegetation and water surfaces, posing a threat to the residents around the farm [54].

In terms of bacterial host families, *Firmicutes* are important ARG hosts and disseminators, likely related to their ability to produce antibiotics as well as their robust stress responses in waste or extreme environments [55,56,57,58,59]. *Firmicutes* and *Bacteroidetes* were found with higher relative abundance in aerosols of animal confinement buildings than in office buildings [60]. These discoveries are worth noting because *Firmicutes* and *Bacteroidetes* are dominant phyla in gut and in feces of livestock animals [61,62,63]. In soil, important potential ARG hosts at the phylum level include *Proteobacteria* and *Bacteroidetes* [49,64,65].

The bacteria that dominate animal intestines and acquire resistance can be excreted into the environment and transferred between environmental matrices, and can even enter the food chain. For example, ARGs that originated in pig manure and wastewater microbiota were identified in soil, vegetation, river water, sediments, and groundwater surrounding a pig farm and were significantly increased by wastewater discharge [66]. There are numerous examples of studies that document this type of effect, although most do not address the question of the fate and mechanism of ARG transmission (Table 1). The references chosen in Table 1 were either newly published in the last three years or have high numbers of citations (above 30).

## 4. Research Methods

### 4.1. Bacterial Culture

Culture methods involve bacterial isolation, purification, and exerting an antimicrobial stress to determine resistance patterns. For example, the resistance patterns of culturable *Campylobacter* from rectal and fecal samples from pigs in France and Sweden indicated that conventional farming practices in France generated greater levels of ARB and ARG transmission than did organic farming practices, however this difference was not apparent in Sweden [78]. The advantage of the culture method is that it usually does not require elaborate laboratory conditions and expensive facilities, while the disadvantages of the method are that it is labor- and time-consuming and the ARG transmission risks may be underestimated [79]. In addition, non-culturable ARB and silent or non-expressed ARGs will be missed, even though the latter can be expressed through gene transfer [14,80]. Common environmental pathogens, such as *L.Listeria monocytogenes*, *Salmonella, E. coli* and *Campylobacter jejuni*, can enter a viable but non-culturable (VBNC) state [81,82,83,84]. In this condition, bacteria cannot produce visible colonies on the culture medium, but are still metabolically active and can be resuscitated and cause infection under suitable conditions. The gradient dilution process commonly used in the culture method is also prone to large errors, resulting in inaccurate results [85].

Despite all of these limitations, the culture method is necessary to investigate the phenotypes of culturable ARB and growth media, such as using Mueller–Hinton (MH) broth or agar to select ARB and determine their minimum inhibitory concentrations (MICs) [49,64]. A complete characterization of environmental ARB requires both culture and molecular-based approaches. When genome sequencing projects are intended to identify novel ARGs or resistance mechanisms, antimicrobial susceptibility testing must be carried out using culture-based method [50].

### 4.2. Molecular Detection

One of the important advantages of molecular detection over the culture-based method is that the former enables the in situ detection and analysis, and thus produces a more comprehensive result. The most frequently employed ARB and ARG detection methods include the polymerase chain reaction (PCR), quantitative PCR (qPCR), high-throughput quantitative real-time PCR (high-throughput qPCR), and high-throughput metagenomic sequencing.

**Figure 1:**
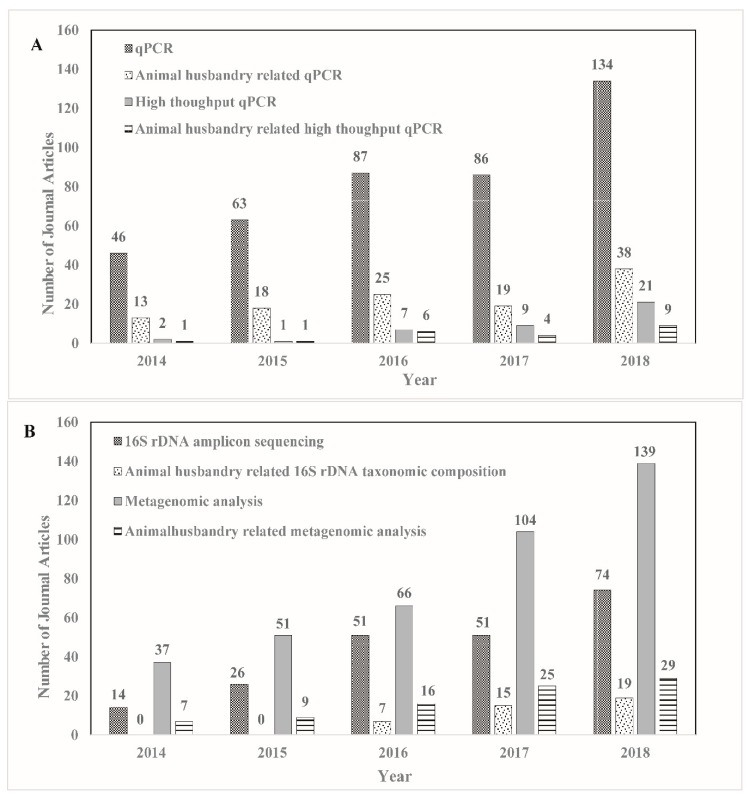
Technical and method journal articles for ARG detection and animal husbandry from the past five years: (**A**) qPCR and high-throughput qPCR; (**B**) 16S rDNA taxonomic composition and metagenomic analysis.

While each molecular detection method has its own limitations, the general disadvantages of molecular detection methods also lie in the incapability to reflect a real transfer risk for certain genes. For example, when analyzing DNA samples, a pair of primers are supposed to amplify the whole abundance of the gene elements that they have been targeted towards, however, not all these gene elements are actually active, while some genes may become “silent” (non-expressing) as their hosts are dormant, inactive, or active without expression of the detected genes while alternative metabolic pathways are expressed. On the other hand, widely existing PCR inhibitors in original samples (such as soil and wastewater), sample processing, and reagents (such as excess salts, phenol, sodium dodecyl sulfate) may cause unreliable results for PCR efficiency. Analyzing total mRNA (metatranscriptomics) can supplement the corresponding gene elements to illustrate how the genes in microorganisms function and how they respond to environmental changes. However, metatranscriptomics is limited by technical, bioinformatic, and conceptual difficulties [86]. The most frequent current usages of molecular methods are listed in Figure 1. 

#### 4.2.1. PCR/qPCR and High-Throughput qPCR

The simple, rapid, and specific characteristics of PCR detection methods make them very practical for pathogen identification and virulence factor and ARG detection, and less prone to false positives compared with bacterial cultures. The potential risks posed by ARGs and pathogenic bacteria in samples can be estimated quantitatively with qPCR and can form the basis for formulating strategies for reducing or eliminating harmful genes [87]. The absolute quantitative method for qPCR can give information about the exact gene abundance in samples before and after treatments. However, this method ignores the difference in extraction efficiency between different DNA samples, and gene copy calculations assume extraction efficiencies are equivalent. In contrast, relative qPCR is a method involving quantification of target genes relative to a reference gene (usually 16S rDNA for bacteria analysis). Relative qPCR does not require taking DNA extraction efficiency errors into account and can be used for data comparisons from different literature sources, regions, and even different environmental samples. The target gene 16S rDNA is also called the normalization gene and is involved in determining gene abundance using absolute qPCR first. This calculation strategy is gaining in popularity because it simultaneously offers absolute and relative abundance of target genes. Researchers should choose among these strategies according to their objectives [44,68,88,89]. 

In general, the PCR and qPCR methods can only amplify a limited number of DNA fragments at a time and mutations and other factors easily lead to difficulties in primer design or inapplicability of primers. Moreover, amplification biases can lead to false negative results due to the presence of PCR inhibitors, or false positive results due to non-specific amplification [77]. Even so, qPCR is still one of the important methods used for quantitative detection of pathogens and ARGs, thanks to its simplicity, accuracy, and low cost.

Since PCR and qPCR are low-throughput methods, usually only a few ARG subtypes are analyzed. In this case, it is important to choose typical or representative genes according to the sample and antimicrobial types. For example, *tetM*, *tetO*, *tetQ*, and *tetW* are frequently encountered tetracycline resistance genes in pig manure. The *tetM* gene can be potentially used as an indicator to monitor manure-borne resistance when the manure is land-applied [4,14,34,90,91]. For erythromycin resistance genes, *ermB*, *ermF*, and *ermG* primers could detect 29%, 14%, and 12% of *erm* sequences, respectively, in swine manure metagenomes [92]. Other types of ARGs that are recommended for detection within wastewater treatment plants (WWTP) or environmental samples include *aph* and *bla*_CTX-M_ [93].

High-throughput qPCR can amplify hundreds of target ARGs in a single run by using gene microarrays, which can improve detection efficiency [46,94,95]. However, the large number of primers that are under the same preset reaction conditions can be problematic, for example discounting the amplification efficiency. High-throughput qPCR cannot overcome the shortcomings of the qPCR technique, except for the high-throughput character, and additionally the microarray supplies are expensive. Therefore, this method is not commonly employed due to the high cost of instruments and supplies, although its use is increasing (Figure 1A).

#### 4.2.2. Bacterial Taxonomic Composition (High-Throughput 16S rDNA Amplicon Sequencing)

Knowing the bacterial taxonomic composition is often necessary to study ARB-related community characteristics in complex environmental matrices, such as manure, soil, and water. ARB community compositions are currently studied using next-generation high-throughput sequencing, such as the Illumina Hiseq and Miseq platforms. Since its development in the 2000s this sequencing technology has matured, and there are now numerous taxonomic (sequence) analysis databases for bacteria, including Green Genes (GG), Silva, and RDP. GG is a commonly used 16S rDNA database (http://greengenes.lbl.gov/) that can sort data from the phylum to the species level, and is the most standardized database, even though the last update was in 2013. Silva (https://www.arb-silva.de/) is used for the analysis of complex bacterial communities in environmental samples. The database updates quickly, but the nomenclature is not standardized. The RDP database can be used directly online, uses standardized nomenclature, but can only annotate to the genus level. Overall, bacterial 16S rDNA amplicon high-throughput sequencing has greatly improved the efficiency of gene sequencing, but only the phylogenetic diversity can be calculated, not profiles of functional genes such as ARGs. Therefore, this technology can only be used to study bacteria and ARG co-occurrence, such as network analysis [96,97]. Moreover, 16S rDNA sequences from many unculturable bacteria cannot even be annotated at the phylum or class levels [98].

#### 4.2.3. Metagenomic Analysis

Metagenomics is the study of genetic material recovered directly from environmental samples, aiming to explore the relation between the microbes and their habitats. Metagenomic sequencing can analyze all the genes in samples and directly carry out bacterial taxonomy and functional gene annotation analyses simultaneously. Its application in ARG and pathogenic gene contamination on farms overcomes the shortcomings of traditional molecular biology methods, which can only analyze a limited number of target genes. This also eliminates problems associated with unsuitable primer design. A thorough introduction of shotgun metagenomic sequencing was previously reviewed [99]. Commonly used metagenomic databases have been previously reviewed in detail [33].

In this technique, the sequences are first subjected to marker gene analysis, binning, and contig assembly, followed by database comparisons using KEGG, COG, MEGAN, GO, MG-RAST, and Swiss-Prot [100]. The prevalently used ARG databases are ARDB, CARD, AGRO, APD3, ARG-ANNOT, ARGs-OAP v2.0, Search Engine for Antimicrobial Resistance (SEAR), DeepARG, INTEGRALL, ISfinder (insertion sequences, IS), and NCBI. Antibiotic Resistance Genes Online (AGRO, https://core.ac.uk/display/7622699) only provides information on vancomycin and β-lactams, while APD3 (http://aps.unmc.edu/AP/) focuses on antimicrobial polypeptides. INTEGRALL [101,102] focuses on integron sequences and gene cassettes, while ISfinder [103] is used for insertion sequence comparisons. The National Center for Biotechnology Information (NCBI) can be used for plasmid gene alignments [104]. Antibiotic Resistance Genes Database (ARDB), maintained by the University of Maryland, was commonly used but is no longer being maintained and has been merged with the CARD database (http://card.mcmaster.ca/home), maintained by McMaster University, Canada. ARG-ANNOT [105], ARGs-OAP v2.0, Search Engine for Antimicrobial Resistance (SEAR) [106], and DeepARG [107] are also commonly used ARG annotation software modules for metagenome sequencing. Their respective characteristics and more ARGs databases are described in detail in a previous review [108]. The database Structured Non-redundant Clean Antibiotic Resistance Genes Database (SNC-ARDB) can be used to annotate ARGs based on metagenome sequencing [73].

Metagenomic methods can help to discover new ARGs in environmental ecosystems [109]. Therefore, metagenomic analysis is defined as an open format approach that is suitable for exploratory discovery studies [109]. A current Web of Science search found that 16S rDNA amplicon and metagenome sequencing projects have steadily increased over the past five years (Figure 1B). The latter approach has been used more, but animal-related studies comprised only a small portion of these studies. Intensive animal husbandry is an important component of ARB pollution so this research focus has room for improvement (Figure 1). 

Metagenomic high-throughput sequencing is also PCR-dependent, and PCR biases can affect the sensitivity and accuracy, for example through exaggerations of dominant taxa or by omitting low abundance taxa. Moreover, metagenomic sequencing often fails to provide sufficient sequencing depth to enrich and simulate the genomes of a single strain, especially in complex environmental samples, such as soil. Binning is one potential solution to this problem, but demands a huge amount of data, greatly increases the cost, and often a sophisticated computer system as well as computer expertise are needed. In addition, if only one class of functional genes (such as ARGs) in the samples are analyzed, and only 0.01–1% of the genes in the environmental samples are related to ARGs, this will result in data excess and waste of cost [109]. The most used sequencing platforms are the Illumina Hiseq and Miseq platforms, while third-generation sequencing with Pacific Biosciences single-molecule real-time (PacBio SMRT) platform is increasingly being used. The latest update for this system enables it to efficiently bin metagenomes into species, strains, and MGEs, which allows a sequence length of up to 3000 bp [33]. For example, metagenomic sequencing combined with the PacBio platform identified basic ARG composition information in dairy cow manure microbiota and found that ARG abundance in dairy cow microbiota was lower than for chicken microbiota. However, PacBio has a lower flow cell throughput, and previously a higher error rate existed when sequencing a continuous long read compared to the Illumina next-generation high-throughput sequencing [110]. Only recently has optimization of circular consensus sequencing (CCS) been believed to improve the accuracy of SMRT sequencing by an average length of 13.5 kb. The new update generates long high-fidelity (HiFi) reads with high accuracy (99.8%), which reach the accuracy level of short next-generation sequencing [111]. If this new method is used, the polishing of Pacbio SMRT sequencing by the next generation sequencing will no longer be needed, although the cost might still be a hinderance to its wide application.

The closed format sequencing methods use functional gene arrays (FGAs). FGAs are DNA microarrays containing probes targeting sequences unique to phylogenetic and functional genes of interest. The most widely used of these is GeoChip, which can analyze tens of thousands of functional genes involved in C, N, S, and P cycling, metal and antimicrobial resistance, and give phylogenetic information using the marker gene *gyrB* [112]. Unlike the traditional high-throughput sequencing approach, GeoChip is PCR-independent. Its sequencing process relies on the hybridization of probes and DNA or RNA to be tested, which enables it to overcome PCR biases and interference caused by sequences derived from plant and animal genomes. This makes it a specific technology that potentially can give quantitative results. The use of GeoChip has grown to include its use in ecosystem ARG detection due to its power in providing sensitive, specific, and quantitative information with good repeatability in water [11,113,114] and extreme environments [115]. However, there are currently no research publications that have used this technology to analyze ARG pollution caused by animal husbandry. Nevertheless, GeoChip is a promising approach for this research field. One of the major drawbacks of GeoChip is that in its construction, the input required must be based upon known gene sequences. Therefore, GeoChip is not able to identify new genes or species and is not suitable for novel explorations. Moreover, as is typical of probe hybridization, since the target sequence must match the probe sequence precisely, homogeneous sequences containing mutations may not attach to the probes, and thereby target gene abundance could be underestimated. The overall advantages and disadvantages of different methods and their suitable applications and scope regarding ARGs are presented in Table 2.

Molecular analysis of ARB and ARGs can reflect the total ARG gene pool, and solves many limitations of cultural-based experiments, including identification of uncultivable bacteria and those that do not survive during manipulation. These analyses have uncovered bacterial mechanisms used to acquire resistance through mutation and horizontal transfer through MGEs, such as plasmids, transposons, -integrons, -phage and insertion sequences. The latter include the discovery of invertons containing promoters regulating ARG expression that can shift to either on or off orientation by flanking inverted repeats. This process can be governed by antimicrobial pressure, indicating that invertons are also genetic elements involved in ARG expression among many bacterial phyla, especially *Bacteroidetes* [116].

#### 4.2.4. Metatranscriptomic Sequencing Technology

Metatranscriptomics is a new sequencing technology that arose from metagenomic sequencing. This technique is used to identify community-wide gene activity profiles from complex environments and ecosystems. However, this type of analysis is still in its infancy and is expensive, but shows great potential as a method to study ARGs at the bacterial community level. The first study of ARGs in environmental microbiota using a multi-omics approach was published in 2017 [106] and has recently been applied to study the intestinal microflora of wild birds, demonstrating the link between human activities and an increase of ARGs in the intestinal tracts of these animals [86]. However, there are no transcriptomics or multi-omics studies of ARGs on animal farms so far. Apart from the cost of research, the complexity of the farm environmental matrices and the unpredictable extraction efficiencies of gene expression products are also important constraints in this field. Future improvements in reagents, extraction methods, and analyzing strategies may help to expand their applications. The primary methods used to study ARG movements on animal farms have primarily utilized qPCR and 16S rDNA taxonomic analysis and metagenomic analysis (Figure 1). However, animal farms are important ARG sources and these new techniques hold promise in identifying how ARGs move through these complex environments and the development of strategies that minimize ARB pollution.

## 5. Conclusions

Due to the extensive use of antimicrobials and intensive animal husbandry in recent decades, ARGs and pathogenic bacteria pollution caused by animal husbandry are increasing. The emergence of antimicrobial-resistant pathogens, especially multidrug-resistant pathogens, are on the rise. There are currently many studies on antimicrobial pollution, ARGs, and pathogens, but the research focus on animal husbandry still needs to be strengthened: (1) At present, this research field is still at the stage of exploring mechanisms, developing detection methods, and calculating preliminary risk assessment. This field has a long way to go to achieve pollution reduction and environmental remediation. (2) Because of cost constraints and technical problems that cannot be overcome in the current biological field, the microbial culture method, qPCR, 16S rDNA sequencing, and even metagenomic sequencing have certain significance, but they cannot fully explain the generation and maintenance mechanisms of ARGs. The kinetic changes, transmission routes, and factors influencing ARG pollution in environmental ecosystems caused by animal husbandry are particularly complex, and it is difficult to evaluate the ecological risks. (3) An understanding of the types and mechanisms of ARG pollution is currently only the tip of the iceberg and there are many gaps of knowledge to be explored. Metatranscriptomics and multi-omics will help to improve knowledge and provide comprehensive assessments of the ecological risk of ARGs. The future use of metagenomics, metatranscriptomics, and multi-omics methods will achieve a more comprehensive analysis and assessment of harmful microbial contamination due to animal husbandry and move toward the goal of reducing and repairing damaged ecosystems caused by animal husbandry.

## Figures and Tables

**Table 1:** Literature related to antimicrobial resistance gene (ARG) environmental dissemination through animal husbandry practices.

Sources	Environmental Matrices	Methods	ARG or Antimicrobial-Resistant Bacteria (ARB) Pollution Results	Reference
Dairy farm	Agricultural soil	Quantitative PCR (qPCR)	Wastewater significantly increased the relative ARG abundance in soil	[67]
Pig, chicken, and cow manure	Soil	High-throughput qPCR and 16S rDNA taxonomic composition	Manure fertilizer significantly increased the ARG abundance	[68]
A semi-intensive beef cattle farm	Soil in feeding and grazing area	qPCR	ARG abundance was negatively correlated with distance from feeding area and abandonment time. Two years after abandonment of cattle farm, ARG pollution still existed	[69]
Pig manure	Soil	qPCR	2×10^−5^ to 0.0374 ARG copies/16S rDNA	[70]
Manure	Soil	PCR	Layering over 15 cm did not distinguish the vertical ARG distribution	[34]
Manure	Soil	qPCR	10^−7^ to 10^−3^ ARG copies/16S rDNA	[71]
Pig manure	Soil	qPCR	Antimicrobial residues and ARGs were found at 60–80 cm depths	[72]
Livestock and others	Multiple environmental matrices	Metagenomic sequencing	Relative ARG abundance: Animal manure > WWTP > river water, soil, and fish pond sediments	[73]
Pig farm	Multiple environmental matrices	qPCR	Antimicrobials and ARGs could penetrate into groundwater, resulting in groundwater pollution	[74]
Pig farms	Aerosols and pig manure	qPCR	The *ermB*, *ermF*, and *tetW* in pig manure >10^9^ copies·g^−1^; ARGs in aerosols were 10^4^ to 10^7^ copies·m^−^	[75]
Poultry farms	Aerosol	PCR	360 strains of *E. coli* were isolated; 47 strains were non-resistant, many were multiply-resistant	[76]
Beef cattle feed yards	Aerosol	qPCR	ARGs were more abundant downwind compared to upwind PM of feed yards	[77]
Pig, layer, and turkey farms	Aerosol	16S rDNA taxonomic composition and qPCR	The abundance of tetracycline ARGs were 10^2^ to 10^6^ copies/ng DNA	[60]

**Table 2:** Comparison of technological methods applied to ARG research.

Method	Advantages	Disadvantages	Application Scope
Culture method	Able to determine the MIC of culturable bacteria and phenotypic changes under antimicrobial selective pressure.	Tedious process; unable for analyzing comprehensive ARG transmission risk on a community level	*Ex situ* phenotypic characterization; selecting and determining MIC of ARB; constructing gene library
PCR/qPCR	Able to determine ARG presence or abundance, offering ARG basic transmission risk	Low-throughput; PCR bias exists; Cannot distinguish between live and dead cell or ARG hosts in a complex community	Determining the presence or abundance of certain interested ARGs with knowing host or obtaining the gene pool
High-throughput qPCR	High-throughput format plus the advantages of qPCR	PCR bias exists; rough abundance data due to the same protocol set for multiple primers	Determines multiple ARGs in environmental microbiota
16S rDNA amplicon sequencing	Allows analysis for bacterial taxa in ecosystems and co-occurrence analysis for bacteria and ARGs	Unable to link ARGs to hosts; PCR bias before sequencing can sometimes influence results	Analyzes bacterial community structure, and potential ARG hosts via co-occurrence analysis
Metagenomic analysis	Allows annotation of all functional genes, making it possible to predict ARG hosts	Poor repeatability and high cost; PCR bias exists; advanced computer system capable of processing huge data sets needed; not sensitive to test low abundance taxa.	Open format analysis allows query of broad characteristics and can identify novel genes; determines the community wide ARG pool
GeoChip	PCR-independent; excellent repeatability and accuracy; high sensitivity enabling detection of low abundance species and genes	Unable to explore novel species or functional genes; potentially underestimating the diversity of microbial taxa and functional genes	Closed format analysis (towards known species and target genes) for phylogenetic and functional genes
